# Supplementary figures and images for: High-Flow Nasal Cannula Oxygen Therapy versus Non-Invasive Ventilation in patients at very high risk for extubating failure: A systematic review of randomized controlled trials
Source: PLoS One. 2024 Apr 3;19(4):e0299693. doi: 10.1371/journal.pone.0299693 (PMC10990192; doi:10.1371/journal.pone.0299693)

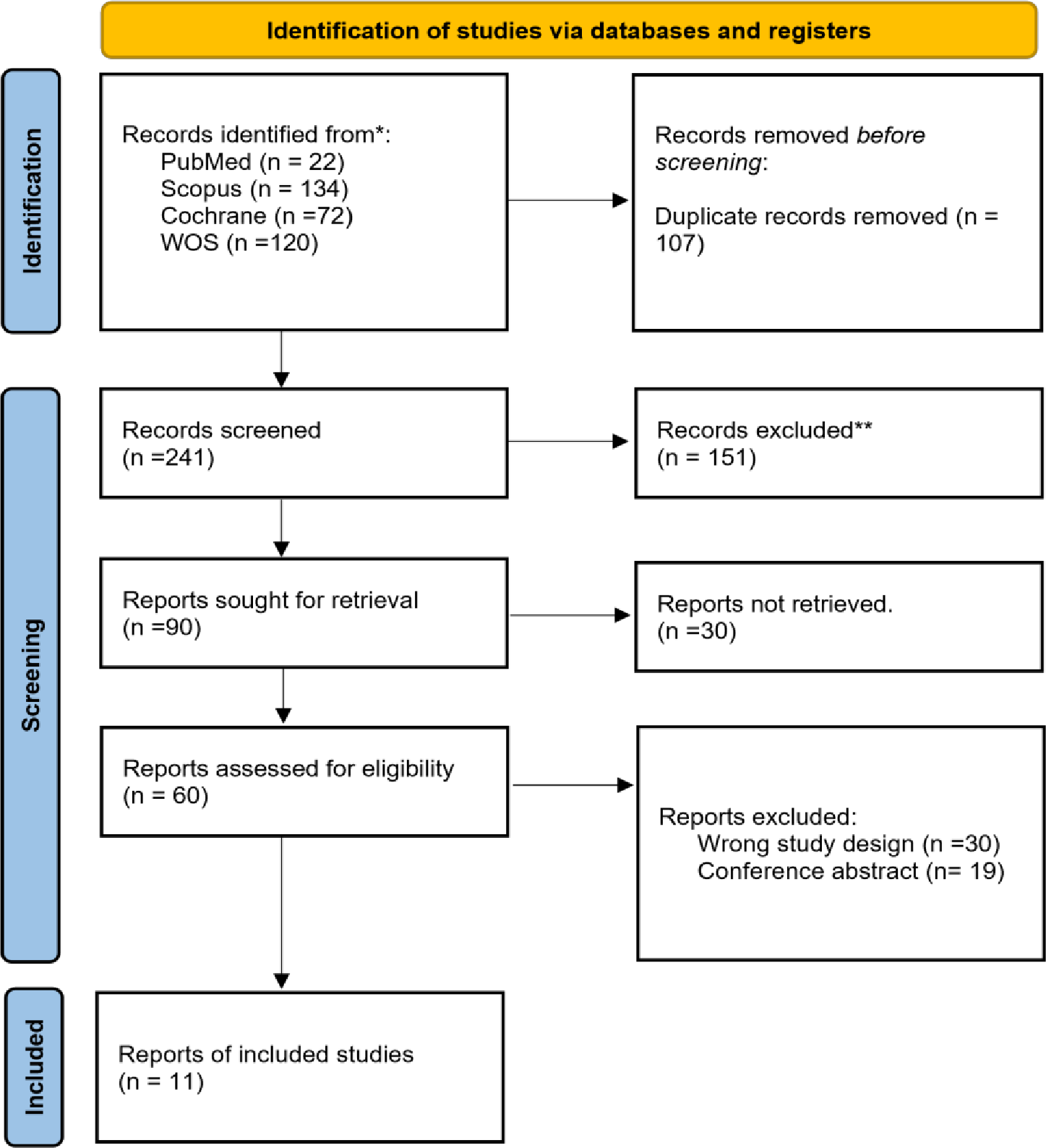

Supplement: S1 Fig — (TIF) [file pone.0299693.s001.tif]

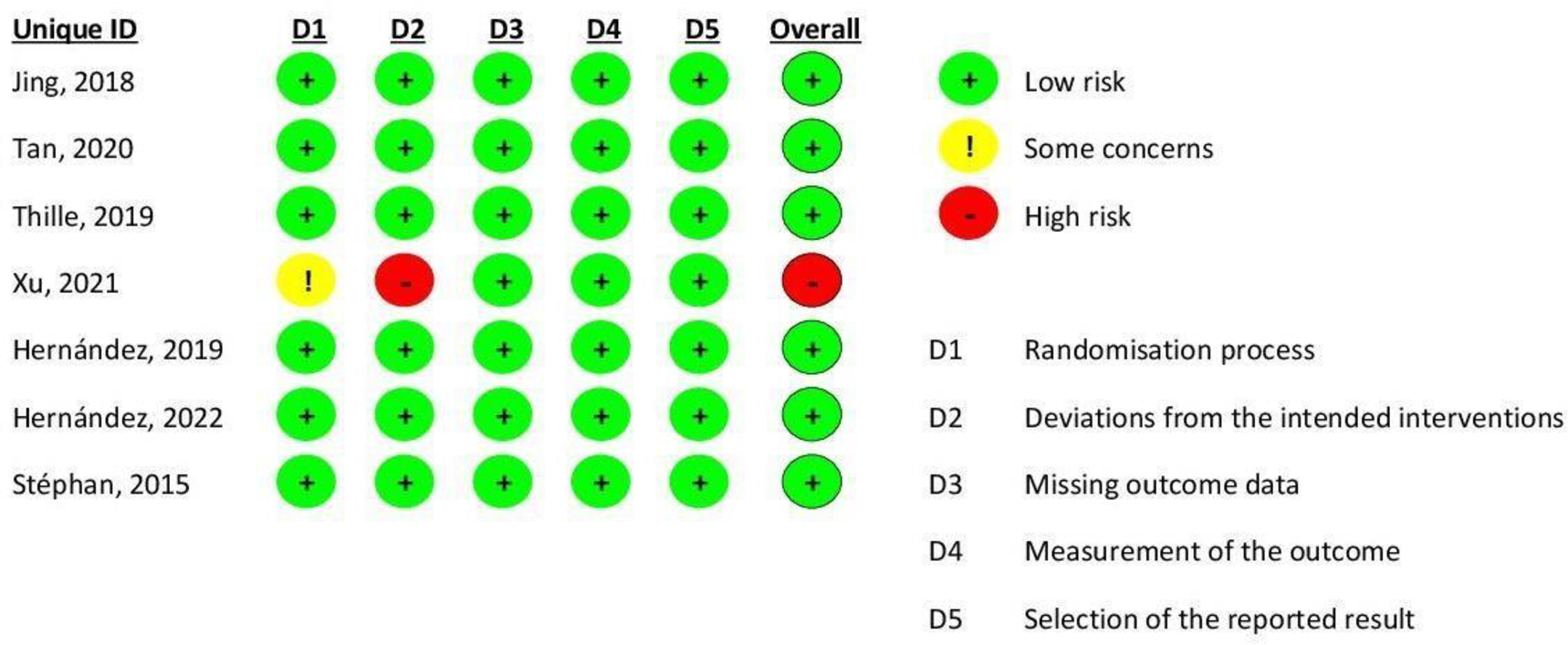

Supplement: S2 Fig — (TIF) [file pone.0299693.s002.tif]
