# Supplementary material for: High-Flow Nasal Cannula Oxygen Therapy versus Non-Invasive Ventilation in patients at very high risk for extubating failure: A systematic review of randomized controlled trials
Source: PLoS One. 2024 Apr 3;19(4):e0299693. doi: 10.1371/journal.pone.0299693 (PMC10990192; doi:10.1371/journal.pone.0299693)
Supplement: S1 Appendix — (DOCX) [file pone.0299693.s003.docx]

**Appendix I. PRISMA Checklist**

| **Section/topic** | **#** | **Checklist item** | **Reported on page #** |
| --- | --- | --- | --- |
| **TITLE** | | |  |
| Title | 1 | Identify the report as a systematic review, meta-analysis, or both. | 1 |
| **ABSTRACT** | | |  |
| Structured summary | 2 | Provide a structured summary including, as applicable: background; objectives; data sources; study eligibility criteria, participants, and interventions; study appraisal and synthesis methods; results; limitations; conclusions and implications of key findings; systematic review registration number. | 2 |
| **INTRODUCTION** | | |  |
| Rationale | 3 | Describe the rationale for the review in the context of what is already known. | 3 |
| Objectives | 4 | Provide an explicit statement of questions being addressed with reference to participants, interventions, comparisons, outcomes, and study design (PICOS). | 4 |
| **METHODS** | | |  |
| Protocol and registration | 5 | Indicate if a review protocol exists, if and where it can be accessed (e.g., Web address), and, if available, provide registration information including registration number. | NA |
| Eligibility criteria | 6 | Specify study characteristics (e.g., PICOS, length of follow-up) and report characteristics (e.g., years considered, language, publication status) used as criteria for eligibility, giving rationale. | 4 |
| Information sources | 7 | Describe all information sources (e.g., databases with dates of coverage, contact with study authors to identify additional studies) in the search and date last searched. | 4 |
| Search | 8 | Present full electronic search strategy for at least one database, including any limits used, such that it could be repeated. | Appendix II |
| Study selection | 9 | State the process for selecting studies (i.e., screening, eligibility, included in systematic review, and, if applicable, included in the meta-analysis). | 5 |
| Data collection process | 10 | Describe method of data extraction from reports (e.g., piloted forms, independently, in duplicate) and any processes for obtaining and confirming data from investigators. | 5 |
| Data items | 11 | List and define all variables for which data were sought (e.g., PICOS, funding sources) and any assumptions and simplifications made. | 5 |
| Risk of bias in individual studies | 12 | Describe methods used for assessing risk of bias of individual studies (including specification of whether this was done at the study or outcome level), and how this information is to be used in any data synthesis. | 5,6 |
| Summary measures | 13 | State the principal summary measures (e.g., risk ratio, difference in means). | NA |
| Synthesis of results | 14 | Describe the methods of handling data and combining results of studies, if done, including measures of consistency (e.g., I^2^) for each meta-analysis. | NA |

**Appendix II. Search strategy**

| **Search strategy item** | **Search strategy details** |
| --- | --- |
| **String of words** | (“High Flow Nasal Cannula” OR “HFNC” OR “High Flow Oxygen Therapy” OR “ High Flow nasal oxygen” OR “High nasal flow” OR “High-Flow nasal cannula oxygen therapy” OR “High Flow oxygen”) AND (“Noninvasive ventilation” OR “Non Invasive Ventilation” OR “NIV”) AND ( “AECOPD” OR “COPD” “high risk for extubation Failure” OR “lung disease” OR “cardiac disease” OR “old age” OR “elderly”) |
| **Databases searched** | PubMed, Scopus, Web of science, and Cochrane library |
| **Inclusion criteria** | P (population): Patients at a high risk for extubating failure  I (intervention/exposure): High-Flow Nasal Cannula Oxygen Therapy C (comparators/comparisons): Non-Invasive Ventilation  O (outcomes): reintubation rate, length of stay, mortality, adverse events, and time to reintubation.  S (study type): Randomized controlled trials (RCTs) |
| **Exclusion criteria** | Non-English studies and study designs that are not RCTs (e.g., observational study designs) |
