# Supplementary material for: High-Flow Nasal Cannula Oxygen Therapy versus Non-Invasive Ventilation in patients at very high risk for extubating failure: A systematic review of randomized controlled trials
Source: PLoS One. 2024 Apr 3;19(4):e0299693. doi: 10.1371/journal.pone.0299693 (PMC10990192; doi:10.1371/journal.pone.0299693)
Supplement: Attachment — Submitted filename: Comments.docx [file pone.0299693.s004.docx]

October, 2023

To the editorial office,

Many thanks for the opportunity to revise and re-submit this manuscript. We have addressed all the reviewers’ comments and outlined below our responses and the amendments made. We thank the reviewers for noting the importance of this manuscript. We hope that you and the reviewers will feel we have addressed the valuable input we have received and look forward to hearing from you regarding publishing our manuscript.

Kind regards,

Dr. Ziyad F Al Nufaiei

**Comments to Reviewers**
1. Is the manuscript technically sound, and do the data support the conclusions?

The manuscript must describe a technically sound piece of scientific research with data that supports the conclusions. Experiments must have been conducted rigorously, with appropriate controls, replication, and sample sizes. The conclusions must be drawn appropriately based on the data presented.

2. Has the statistical analysis been performed appropriately and rigorously?

Reviewer #1: Yes

Reviewer #2: N/A

3. Is the manuscript presented in an intelligible fashion and written in standard English?

PLOS ONE does not copyedit accepted manuscripts, so the language in submitted articles must be clear, correct, and unambiguous. Any typographical or grammatical errors should be corrected at revision, so please note any specific errors here.

Reviewer #1: Yes

Reviewer #2: No

4. Review Comments to the Student

Reviewer #1: Well written systematic review and meta analysis of RCT comparing efficacy of NIV vs HFNC in recently extubated patients. It’s a very interesting and important topic and of great importance in current clinical practice . As mentioned HFNC is extensively used in hospitals and much preferred by patients because of convenience and comfort to use.
Points that could have included on discussion:
How HFNC helps in hypercapnia
The study still leave the readers in an inconclusive state that which is more beneficial. I think it’s better to conclude that a combination of both is more effective that one Alone .

Reviewer #2: Abstract:
Background: Line 2 in background section I suggest changing “them” to patients

Reply: changed

Line 3, I suggest changing “some” to few or give an appropriate measure of this problem. Some patients do not respond well to it, is very generic.

Reply: changed to few

Methods: Line 5 please clearly write length of ICU stay or length of hospital stay?

Reply: edited as both ICU and hospital stay

Results: Line 1 and 2, please indicate did you only select RCTs of COPD patients with high risk of reintubation or all RCTs of patients with high risk for re-intubation? In the background section line 5 you have mentioned you aimed to study HFNC versus NIV among previously intubated COPD patients.

Reply: edited as the study included COPD patients only

Introduction: Line 1 and 2, Ref [1] this was a pilot study done in 10 COPD patients to see hemodynamic effects of spontaneous breathing trial on pulmonary artery occlusion pressure. Patients were treated with dobutamine infusion followed by Levosemindan to assess the hemodynamic benefit of both infusions. Reference provided here does not match by statement made by authors. I suggest providing a different reference for the statement made in line one and two or no reference needed for this statement.

Reply: I have changed the reference

Line 4- I will suggest changing “deteriorating respiratory mechanics” to deteriorating hemodynamics as study cited here studied hemodynamic effects of spontaneous breathing trials in COPD patients. This study did not evaluate respiratory mechanics.

Reply: changed

Line 5-6 Ref [2], this study evaluated the attributable morbidity and mortality of ventilator-associated pneumonia (VAP) in intensive care unit (ICU) patients. Findings noted increase ICU length of stay and trend towards increased mortality with VAP. This study did not evaluate adverse effects of prolonged mechanical ventilation. This reference is not the most appropriate to reflect the statement made here. Please consider changing reference, suggested reference would be: Coplin WM, Pierson DJ, Cooley KD, et al. Implications of extubation delay in brain-injured patients meeting standard weaning criteria. Am J Respir Crit Care Med 2000;161:1530-6.

Reply: I have changed all references

Line 9, 10, 11: Ref [5], this study evaluated home NIV monitoring in COPD patients, not a appropriate reference for NIV patients immediately post extubation.

Reply: I have checked the reference, and proper reference is used

Ref [6] appropriately cited and supports the statement made by authors.

Reply: Agree

Line 13, 14, 15 Ref [7], this study evaluated utility of Heliox in acute COPD exacerbation. Results noted Heliox improves respiratory acidosis, encephalopathy, and the respiratory rate more quickly than air/O2 but does not prevent NIV failure. I suggest using a more appropriate reference to support the statement made here.

Reply: This reference was removed

Ref [8] Multinational observational cohort study recording trends of mechanical ventilation from 1998 to 2004 does not support statement made here. Please consider adding another reference supporting statement made.
Reply: I have updated the reference

Line 15 and 16, NIV associated with difficulty is aspiration is an incorrect statement. Data supports NIV associated with increased aspiration risk. Please make appropriate corrections here. Ref [9] appropriately cited.

Reply: the reference has been updated

Ref [10] excellent reference to support the use of high flow oxygen in acute hypoxemic respiratory failure. FLORALI RCT studying high flow oxygen with standard of care and NIV in acute hypoxemic respiratory failure (non-hypercapnic patients). High flow oxygen group showed significantly lower ventilator free days and increased 90-day survival.

Reply: Agree

Methods:
Inclusion criteria:
Line 2 and 3 please make it clear if you only studied RCTs with COPD patients?

Reply: Updated by adding COPD

Search Strategy: Line 3 mentions appendix II details the search term but there is no appendix shown in manuscript.

Reply: added

Screening, data extraction and quality assessment: Line 9 Ref [20] listed as type of biases but this is a study evaluating HFNC versus NIPPV outcomes post extubation. Please cite this reference accordingly.
Reply: the reference has been updated

Results: Line 3 you have mentioned 30 articles were removed as you were unable to retrieve those articles. Please explain this in more detail as important studies may have been missed in this analysis (risk for bias in analysis).

Reply: Thank you for your comment. You point is valid. The exclusion of an additional 30 articles from the study was necessary due to study design. As a result, we conducted our study based on the articles that were successfully retrieved and accessible, ensuring the reliability and completeness of the data used in our research. To be more transparent about this and make it clearer to the readers, we included explanation about this as part of our strengths and limitations subheading within the discussion section

Study characteristics: Line 5, you have mentioned full texts were not available for 3 abstracts (18), (21), and (26). Do these numbers mean references listed in manuscript or these numbers reflect something else? Ref 18 is a study on assessing biases in RTCs. Ref 21 and 26 are open access articles easily available. Please explain?

Reply: The references have been updated

Table 1: There is significant heterogeneity in studies listed in this table. I will suggest authors make a separate column to indicate potential weaknesses or biases of individual studies mentioned in table.

Reply: Heterogeneity is not important here as the we didn’t conduct a meta-analysis

Please also consider adding columns in your table including adverse events, and time to reintubation as you have listed these as your study outcomes.

Reply: Added
Ref [20], study from China reported the rate of tracheal re-intubation within 7 days in the HFNC group was significantly higher than that in the NIPPV group [35.0% (7/20) vs. 4.2 % (1/24), P < 0.05]. You have mentioned re-intubation rate were not reported in this study. Please make corrections in your table.

Reply: updated
Table 1 Ref [21], column 6 the reintubation needs correction. In this study, 21 [23.3%] patients NIV group vs 35 [38.8%] patients HFNC group. I would suggest making this change to the table. Too many numbers in this column are confusing.

Reply: updated

Table 1 Ref [22] column 6 please indicate re-intubation rates in both groups (HFNC vs NIV). In this study included total 604 patients, 66 patients (22.8%) in the high-flow group vs 60 (19.1%) in the NIV group required re-intubation.

Reply: updated

Table 1 Ref [23] small pilot studies, interpretation is limited from this RCT. Data showing in abstract reflects only 48 hours post extubation.

Reply: Agree
Table 1 Ref [24] column 6 please indicate re-intubation rates were similar in both groups. In this study, 6(13.6) patients in HFNC group and 6(14.29) patients in NIV group required re-intubation. This was shown in table 2 of this article.

Reply: updated

Table 1 Ref [25] column 6 needs correction. In this French study, reintubation was performed in 57 patients with BiPAP (13.7%) and 58withhigh-flownasal oxygen therapy (14.0%) (P = .99).

Reply: updated

Table 1 reference [27] this is a meta-analysis evaluating HFNC in type II respiratory failure. I would suggest removing this meta-analysis from your systematic review. This did not evaluate the utility of HFNC post extubation. This study doesn’t match with your inclusion criteria please explain why study was included in analysis.

Reply: the reference has been updated

Table 1 [Ref 29] agree with authors.

Quality assessment and risk of bias:
Reintubation: Line 1 (19) please explain what this number indicates as findings written in line 1-4 are Ref [21]. Line 1-4 need corrections.
Reply: updated

Line 4 (20) please explain what this number indicates as study cited here is not Ref [20]. Please cite reference at the right place in the article.
Reply: updated

High-Flow Nasal Cannula Oxygen Therapy versus Non-Invasive Ventilation
Multiple mistakes in citing the reference. Reference number cited does not match with reference listed. Please make these corrections so this section can be reviewed appropriately.
Reply: updated

Discussion:
Paragraph 2, Line 6 and 7 mentioned: To date, o meta-analysis or systematic review specifically focused on the role of NIV post extubation. This statement made here by authors does not support the existing literature. You have cited the following meta-analysis and systematic review at Ref [46] contradicting statement made here.

Huang HW, Sun XM, Shi ZH, et al. Effect of High-Flow Nasal Cannula Oxygen Therapy Versus Conventional Oxygen Therapy and Noninvasive Ventilation on Reintubation Rate in Adult Patients After Extubation: A Systematic Review and Meta-Analysis of Randomized Controlled Trials. J Intensive Care Med. 2018;33(11):609-623. doi:10.1177/0885066617705118

Reply: the mentioned references are not meta-analyses

Please review references cited in the discussion section and references listed. There are multiple mismatches in listings.

Reply: updated

Strengths and limitations:
Comprehensive review of literature is the strength of this systematic review.
Weaknesses authors did not evaluate all the outcomes listed in the introduction section of the article. There is significant heterogeneity in the studies listed in this systematic review. Authors have not explored study biases in this systematic review. Studies with low sample size and significant biases may have been overrepresented in this systematic review. RCTs with high quality of evidence may have been underrepresented.

References:
Ref [1] Inappropriately cited

Reply: updated

Ref [2] his study evaluated the attributable morbidity and mortality of ventilator-associated pneumonia (VAP) in intensive care unit (ICU) patients . Findings noted increase ICU length of stay and trend towards increased mortality with VAP. This reference is not the most appropriate to reflect the statement made by authors.

Reply: updated

Ref [15] and [16] are listed in the reference list but not cited anywhere in the manuscript.

Reply: updated
