# Supplementary material for: High-Flow Nasal Cannula Oxygen Therapy versus Non-Invasive Ventilation in patients at very high risk for extubating failure: A systematic review of randomized controlled trials
Source: PLoS One. 2024 Apr 3;19(4):e0299693. doi: 10.1371/journal.pone.0299693 (PMC10990192; doi:10.1371/journal.pone.0299693)
Supplement: Attachment — Submitted filename: Response letter to reviewers comments.docx [file pone.0299693.s005.docx]

07^th^ December 2023

To the editorial office of PLOS ONE Journal,

Many thanks for the opportunity to revise and re-submit this manuscript. We have addressed all the reviewers’ comments and outlined below our responses and the amendments made. We thank the reviewers for noting the importance of this manuscript. We hope that you and the reviewers will feel we have addressed the valuable input we have received and look forward to hearing from you regarding publishing our manuscript.

Kind regards,

Ziyad F Al Nufaiei, PhD, RRT-NPS, CPFT

Respiratory Therapy Department

College of Applied Medical Sciences-Jeddah

King Saud bin Abdulaziz University for Health Sciences

Research office, King Abdullah International Medical Research Center

Mail Code 6610

P.O.Box.9515 Jeddah, 21423	Ext. 46125

E-mail: nufaieiz@ksau-hs.edu.sa

**Reviewers’ Comments:**

1. **Well written systematic review and meta analysis of RCT comparing efficacy of NIV vs HFNC in recently extubated patients. It’s a very interesting and important topic and of great importance in current clinical practice . As mentioned HFNC is extensively used in hospitals and much preferred by patients because of convenience and comfort to use.
   Points that could have included on discussion:
   How HFNC helps in hypercapnia
   The study still leave the readers in an inconclusive state that which is more beneficial. I think it’s better to conclude that a combination of both is more effective that one Alone.**

Thank you so much for your positive comments on the systematic review. That is very appreciated.

With regards to your feedback on the discussion and conclusion sections, your comments have been taken into consideration and further details have been added [lines 194-205] and [lines 232 – 239].

2. **Abstract: Background: Line 2 in background section I suggest changing “them” to patients**

Thank you for noting this. It has been amended as advised.

3. **Line 3, I suggest changing “some” to few or give an appropriate measure of this problem. Some patients do not respond well to it, is very generic.**

You have a good point. I therefore, amended the sentence to make it more clear to “while many patients respond well to NIV, a significant proportion” *- which might range from a small to a relatively large percentage –* “may not respond as favorably”.

4. **Methods: Line 5 please clearly write length of ICU stay or length of hospital stay?**

Thanks for your comment. As our outcome related to this included both or either, I made the appropriate amendments to the text in the methods of the abstract to make this clearer.

5. **Results: Line 1 and 2, please indicate did you only select RCTs of COPD patients with high risk of reintubation or all RCTs of patients with high risk for re-intubation? In the background section line 5 you have mentioned you aimed to study HFNC versus NIV among previously intubated COPD patients.**

Thanks for highlighting this. Yes, you are correct. Our review is only on COPD patients and I added this to the results of the abstract as advised.

6. **Introduction: Line 1 and 2, Ref [1] this was a pilot study done in 10 COPD patients to see hemodynamic effects of spontaneous breathing trial on pulmonary artery occlusion pressure. Patients were treated with dobutamine infusion followed by Levosemindan to assess the hemodynamic benefit of both infusions. Reference provided here does not match by statement made by authors. I suggest providing a different reference for the statement made in line one and two or no reference needed for this statement.**

Reference [1] has been amended for a more accurate reference based on the background information provided at the beginning of the introduction section (lines 35-38).

7. **Line 4- I will suggest changing “deteriorating respiratory mechanics” to deteriorating hemodynamics as study cited here studied hemodynamic effects of spontaneous breathing trials in COPD patients. This study did not evaluate respiratory mechanics.**

Thank you for noting this. This has been amended in line 39 of the introduction section as suggested.

8. **Line 5-6 Ref [2], this study evaluated the attributable morbidity and mortality of ventilator-associated pneumonia (VAP) in intensive care unit (ICU) patients. Findings noted increase ICU length of stay and trend towards increased mortality with VAP. This study did not evaluate adverse effects of prolonged mechanical ventilation. This reference is not the most appropriate to reflect the statement made here. Please consider changing reference, suggested reference would be: Coplin WM, Pierson DJ, Cooley KD, et al.** **Implications of extubation delay in brain-injured patients meeting standard weaning criteria. Am J Respir Crit Care Med 2000;161:1530-6.**

Thank you for your comment. The reference you suggested has been included as reference [2].

9. **Line 9, 10, 11: Ref [5], this study evaluated home NIV monitoring in COPD patients, not a appropriate reference for NIV patients immediately post extubation.**

Thanks for highlighting this. Reference [5] has been updated to reflect on the sentence provided.

10. **Ref [6] appropriately cited and supports the statement made by authors.**

Thank you for your positive comment.

**Line 13, 14, 15 Ref [7], this study evaluated utility of Heliox in acute COPD exacerbation. Results noted Heliox improves respiratory acidosis, encephalopathy, and the respiratory rate more quickly than air/O2 but does not prevent NIV failure. I suggest using a more appropriate reference to support the statement made here. Ref [8] Multinational observational cohort study recording trends of mechanical ventilation from 1998 to 2004 does not support statement made here. Please consider adding another reference supporting statement made. Line 15 and 16, NIV associated with difficulty is aspiration is an incorrect statement. Data supports NIV associated with increased aspiration risk. Please make appropriate corrections here. Ref [9] appropriately cited. Ref [10] excellent reference to support the use of high flow oxygen in acute hypoxemic respiratory failure. FLORALI RCT studying high flow oxygen with standard of care and NIV in acute hypoxemic respiratory failure (non-hypercapnic patients). High flow oxygen group showed significantly lower ventilator free days and increased 90-day survival.**

Thank you for highlighting the points about the references. References and literature explored has been updated in all the sections advised by the reviewer and amendments have been made accordingly.

11. **Methods: Inclusion criteria: Line 2 and 3 please make it clear if you only studied RCTs with COPD patients?**

Thank you for your feedback. To make it clear to the readers, I’ve included “COPD” in line 72 of the methods section.

12. **Search Strategy: Line 3 mentions appendix II details the search term but there is no appendix shown in manuscript.**

Thank you for this. We have a sentence in the search strategy section “Appendix II details the search terms used and PICO framework” in line 81-81. Appendices are attached separately as supplementary materials.

13. **Screening, data extraction and quality assessment: Line 9 Ref [20] listed as type of biases but this is a study evaluating HFNC versus NIPPV outcomes post extubation. Please cite this reference accordingly.**
    The reference has been updated as advised.
14. **Results: Line 3 you have mentioned 30 articles were removed as you were unable to retrieve those articles. Please explain this in more detail as important studies may have been missed in this analysis (risk for bias in analysis).**

Thank you for your comment. You point is valid. In the manuscript, we mentioned that these articles were removed as they did not meet our inclusion criteria (i.e., inappropriate study designs). We acknowledge that excluding these articles poses a potential risk for bias. To mitigate this, we ensured that our search strategy was comprehensive, covering multiple databases to capture as many relevant studies as possible. Also, where possible, we attempted to contact authors of these inaccessible articles to obtain the full texts. In addition, we reviewed the reference lists of the included studies to identify any potentially relevant studies that might have been missed. Nonetheless, to be more transparent about this and make it clearer to the readers, we included explanation about this as part of our strengths and limitations subheading within the discussion section

15. **Study characteristics: Line 5, you have mentioned full texts were not available for 3 abstracts (18), (21), and (26). Do these numbers mean references listed in manuscript or these numbers reflect something else? Ref 18 is a study on assessing biases in RTCs. Ref 21 and 26 are open access articles easily available. Please explain?**

Apologies for this as the references were wrongly placed. Now, the references have been amended as shown in the main manuscript line 112, ref [16 – 18].

16. **Table 1: There is significant heterogeneity in studies listed in this table. I will suggest authors make a separate column to indicate** **potential weaknesses or biases of individual studies mentioned in table.**

We appreciate your suggestion to include a column indicating potential weaknesses or biases of individual studies in Table 1. However, our systematic review was designed to provide a comprehensive overview of the existing literature on the subject without conducting a meta-analysis. The primary aim was to summarize and synthesize the findings of the included studies rather than critically appraising individual study quality or bias in detail. Including a column for potential weaknesses or biases could imply a level of detailed critical appraisal akin to a meta-analysis. Since our review does not include a meta-analysis, such detailed critique of individual studies goes beyond the scope of our methodology. Nonetheless, we acknowledge the heterogeneity among the included studies, which is an inherent aspect of systematic reviews. This heterogeneity is discussed in the discussion section within our limitations, where we address how it might affect the interpretation of our findings line 210-212, page 4).

We believe that this approach maintains the integrity of our systematic review and appropriately addresses the issue of study heterogeneity while staying within the bounds of our review's objectives and methodology.

17. **Please also consider adding columns in your table including** **adverse events, and time to reintubation as you have listed these as your study outcomes.**

Thanks for highlighting this. These two columns has been added to Table 1.

**Ref [20], study from China reported the rate of tracheal re-intubation within 7 days in the HFNC group was significantly higher than that in the NIPPV group [35.0% (7/20) vs. 4.2 % (1/24), P < 0.05]. You have mentioned re-intubation rate were not reported in this study. Please make corrections in your table. Table 1 Ref [21], column 6 the reintubation needs correction. In this study, 21 [23.3%] patients NIV group vs 35 [38.8%] patients HFNC group. I would suggest making this change to the table. Too many numbers in this column are confusing. Table 1 Ref [22] column 6 please indicate re-intubation rates in both groups (HFNC vs NIV). In this study included total 604 patients, 66 patients (22.8%) in the high-flow group vs 60 (19.1%) in the NIV group required re-intubation. Table 1 Ref [23] small pilot studies, interpretation is limited from this RCT. Data showing in abstract reflects only 48 hours post extubation. Table 1 Ref [24] column 6 please indicate re-intubation rates were similar in both groups. In this study, 6(13.6) patients in HFNC group and 6(14.29) patients in NIV group required re-intubation. This was shown in table 2 of this article.Table 1 Ref [25] column 6 needs correction. In this French study, reintubation was performed in 57 patients with BiPAP (13.7%) and 58withhigh-flownasal oxygen therapy (14.0%) (P = .99). Table 1 reference [27] this is a meta-analysis evaluating HFNC in type II respiratory failure. I would suggest removing this meta-analysis from your systematic review. This did not evaluate the utility of HFNC post extubation. This study doesn’t match with your inclusion criteria please explain why study was included in analysis.**

Thank you for your valuable feedback. All references mentioned above has been updated as advised and suggested.

18. **Quality assessment and risk of bias:
    Reintubation: Line 1 (19) please explain what this number indicates as findings written in line 1-4 are Ref [21]. Line 1-4 need corrections.**

This has been updated.

19. **Line 4 (20) please explain what this number indicates as study cited here is not Ref [20]. Please cite reference at the right place in the article.**

Thank you. This has been amended and the reference updated.

20. **High-Flow Nasal Cannula Oxygen Therapy versus Non-Invasive Ventilation
    Multiple mistakes in citing the reference. Reference number cited does not match with reference listed. Please make these corrections so this section can be reviewed appropriately.**
    Thank you. This has been amended and the reference updated accordingly.
21. **Discussion:
    Paragraph 2, Line 6 and 7 mentioned: To date, no meta-analysis or systematic review specifically focused on the role of NIV post extubation. This statement made here by authors does not support the existing literature. You have cited the following meta-analysis and systematic review at Ref [46] contradicting statement made here.**

**Huang HW, Sun XM, Shi ZH, et al. Effect of High-Flow Nasal Cannula Oxygen Therapy Versus Conventional Oxygen Therapy and Noninvasive Ventilation on Reintubation Rate in Adult Patients After Extubation: A Systematic Review and Meta-Analysis of Randomized Controlled Trials. J Intensive Care Med. 2018;33(11):609-623. doi:10.1177/0885066617705118**

Thank you for your insightful comment regarding the citation of Huang et al.'s systematic review and meta-analysis. Upon re-evaluation, I recognize that the cited study does compare high-flow nasal cannula oxygen therapy with conventional oxygen therapy and noninvasive ventilation (NIV) in terms of reintubation rates post-extubation. However, I would like to clarify that the focus of my statement in the manuscript was on the lack of systematic reviews or meta-analyses specifically targeting the use of NIV in high-risk patients for extubation failure. While Huang et al.’s work is indeed relevant in the broader context of post-extubation interventions, it does not explicitly concentrate on the high-risk patient subgroup. This distinction is critical in our study's context and warrants further investigation. Nonetheless, as your comment is valid and the readers might mis-interpret this, I amended the sentence as shown in lines 156-158.

22. **Please review references cited in the discussion section and references listed. There are multiple mismatches in listings.**

Thank you for pointing this out. The references has been updated.

23. **Strengths and limitations:
    Comprehensive review of literature is the strength of this systematic review.
    Weaknesses authors did not evaluate all the outcomes listed in the introduction section of the article. There is significant heterogeneity in the studies listed in this systematic review. Authors have not explored study biases in this systematic review. Studies with low sample size and significant biases may have been overrepresented in this systematic review. RCTs with high quality of evidence may have been underrepresented.**

Thank you so much for your comments. The whole section has been amended to reflect on the points that you mentioned as shown in line 207-224 of the discussion section.

24. **References:
    Ref [1] Inappropriately cited. Ref [2] his study evaluated the attributable morbidity and mortality of ventilator-associated pneumonia (VAP) in intensive care unit (ICU) patients . Findings noted increase ICU length of stay and trend towards increased mortality with VAP. This reference is not the most appropriate to reflect the statement made by authors. Ref [15] and [16] are listed in the reference list but not cited anywhere in the manuscript.**

Thank you for noting this. All of the references have been double checked and updated throughout the manuscripts as appropriate and suggested.
